# Image guidance: past and future of radiotherapy

**DOI:** 10.1007/s00117-019-0573-y

**Published:** 2019-07-25

**Authors:** H. Herrmann, Y. Seppenwoolde, D. Georg, J. Widder

**Affiliations:** Dept of Radiotherapy, Medical University of Vienna, Währinger Gürtel 18–20, 1090 Vienna, Austria

**Keywords:** Magnetic resonance imaging, Computed tomography, Three-dimensional imaging, Four-dimensional CT, Radiation dosage, Magnetresonanzbildgebung, Computertomographie, Dreidimensionale Bildgebung, Vierdimensionale Computertomographie, Bestrahlungsdosis

## Abstract

Image guidance has been playing a decisive role throughout the history of radiotherapy, but developments in 3D-and 4D imaging data acquisition using computed tomography (CT), magnetic resonance imaging (MRI), and positron emission tomography (PET) have significantly boosted the precision of conformal radiotherapy. An overarching aim of radiotherapy is conforming the treatment dose to the tumor in order to optimally limit a high radiation dose outside the target. Stereotactic, intensity modulated, and adaptive radiotherapy are all largely based on appropriately using imaging information both before and during treatment delivery using on-board imaging devices. While pretreatment imaging for planning has reached a very high level in the past two decades, the next step will be to further refine and accelerate imaging during treatment delivery, resulting in adaptation of the dose fluence during a patient’s treatment in various scenarios, some of which are discussed in this article.

## Zusammenfassung

In der Geschichte der Radiotherapie hat Bildführung immer eine große Rolle gespielt. Entwicklungen im Bereich von 3‑D- und 4‑D-Bilddaten, die mittels Computertomographie (CT), Magnetresonanztomographie (MRT) und Positronenemissionstomographie (PET) gewonnen wurden, haben die Präzision der konformalen Radiotherapie deutlich gesteigert. Ein übergeordnetes Ziel der Technik der Radiotherapie besteht in der Konformation der Behandlungsdosis auf den Tumor, um hohe Dosen außerhalb des Zielvolumens optimal zu begrenzen. Die stereotaktische, intensitätsmodulierte und adaptive Radiotherapie beruhen alle auf einer angemessenen Verwendung von Bildinformation sowohl im Planungsprozess als auch während der Therapie; dabei kommt direkt mit dem Bestrahlungsgerät verbundene Bilderzeugung zum Einsatz. Während die Bildgebung im Rahmen der Therapieplanung in den vergangenen 2 Dezennien einen sehr hohen Standard erreicht hat, wird weiterer Fortschritt in Entwicklungen der Bildgebung während der Radiotherapie liegen; dadurch kann die Anpassung der Dosisfluenz während der Behandlung in verschiedenen Situationen erreicht werden. Einige solcher Beispiele werden hier besprochen.

Relatively early in the history of radiotherapy (RT), X‑ray images were employed to guide treatment [1]. For a long period of time, simulation of the actual radiation therapy by means of fluoroscopy and kilovolt X‑ray images acquired from the patient in treatment position determined the volume to be treated. Treatment fields were thus typically oriented at bony landmarks, and tumors as well as healthy tissues were translated into these X‑ray images that represented the treatment fields. In this process, information about anatomical relationships (e.g., the spinal cord residing within the spinal canal of the vertebral bodies; mediastinal lymph nodes identified by projecting them to vertebral bodies and the trachea) and information about tumor localization retrieved from clinical examination and anatomical knowledge were correlated and taken into account in the process of defining radiation portals. These radiation portals were therefore highly standardized and treatment was often characterized via “treatment fields.” The advent of computerized tomography (CT) revolutionized radiotherapy, in that it became possible to directly visualize the tumor and organs to be spared from radiation: Treatment beams could now be individually oriented for a given patient with their tumor, which marked the start of the era of 3D-conformal radiotherapy—conforming the treatment dose to the tumor based on 3D images.

**Fig. 1:**
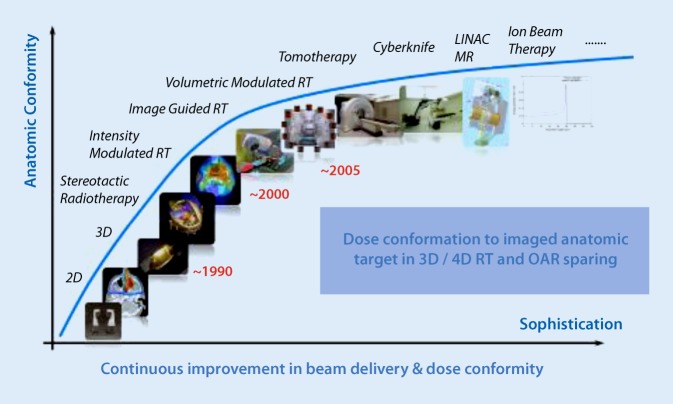
Improvement in radiotherapy (*RT*) during the past three decades. *MR* magnetic resonance, *OAR* organ at risk

Over the past decades, precision and consequently efficacy in radiation oncology have increased considerably. This achievement is firstly due to dramatic improvements in treatment planning, enabling the technology of fluence (intensity) modulated beam delivery using advanced computation algorithms, both for photon-based as well for particle-based radiotherapy. In intensity-modulated radiotherapy (IMRT), the treatment is modulated to deliver lower doses to organs at risk (OARs) adjacent to the target volume while escalating the dose to the target volume [2]. Typically, a homogeneous dose is prescribed to the planning target volume (PTV) encompassing the tumor. A more advanced RT strategy, developing further the concept of dose intensification to the volume requiring the highest radiation dose, could be a combination of anatomical imaging with functional imaging to specifically boost less perfused (hypoxic) or highly proliferating areas within this target volume [3]. Secondly, these developments are inseparable from progress in imaging technology. X‑ray-based imaging was taken on-board the treatment unit itself, so that in-room image guidance presently is the standard of practice in radiation oncology [4, 5]. More recently, image-guided radiotherapy (IGRT) increasingly includes time as a fourth dimension in treatment planning and delivery (Fig. 1), thus accounting for any movements during the course of RT.

Influenced and motivated by the IGRT standards in photon beam therapy, the integration of advanced X‑ray imaging (e.g., cone-beam computed tomography, CBCT) in beam lines and gantries has just started in proton and ion beam therapy. Despite all the advancements achieved during the past few decades, it still remains challenging to detect, assess, delineate, and track the tumor with the ultimate aim of further reducing safety margins in order to hit the tumor and spare healthy tissues as much as possible. First, the soft tissue contrast of CT and especially of on-board CBCT is limited; second, intense X‑ray imaging results in increased radiation dose to usually un-exposed healthy tissues [6–8]. This additional imaging-associated radiation dose in IGRT needs to be assessed and minimized as it may be linked to an increased probability of secondary cancer (see Fig. 5).

In this article, we discuss a few recent developments in IGRT. We show that employing a range of imaging modalities in the planning process as well as at delivery of RT, combined with algorithms optimizing the radiation dose distributions, has played a critical role in making RT one of the medical disciplines most driven by advances in imaging technology.

## Image-based adaptive RT

Routine clinical RT is typically based on a CT scan obtained during the treatment planning phase, which is often co-registered with pretreatment magnetic resonance imaging (MRI) or positron emission tomography (PET) scans in addition. The dose distribution is calculated and optimized based on the organ configuration that was present on the day of scanning. To ensure dose coverage of the tumor, safety margins are created around the target volumes to account for patient repositioning uncertainties and/or tumor motion [9, 10]. However, during the course of RT, both the tumor and healthy surrounding organs are variable in size and position because of anatomical changes between fractions (inter-fraction variations), or even during beam delivery within one treatment fraction (intra-fraction variations).

For tumors at different locations in the body, the origin and extent of motion can vary [11]. For patients with lung cancer, the tumor moves with breathing (intra-fraction motion); for patients with a tumor in the pelvic area, the position is dependent on bladder and bowel filling (inter- and sometimes intra-fraction motion). Patients with a tumor in the head and neck region often lose weight during treatment, resulting in a more gradual inter-fraction change. Furthermore, during the course of treatment, the tumor may become smaller or the tumor biology might change.

**Fig. 2:**
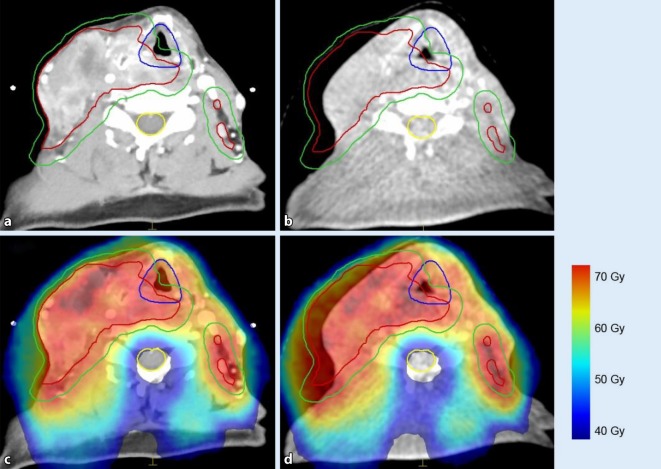
Image-guided radiotherapy (RT) for head and neck cancer. Planning computed tomography (**a**) overlaid with dose (**c**); same patient in week 5 of treatment (**b**, **d**). Although the treated lymph nodes evidently regressed (**b**), dose coverage is maintained even without adaptation of the treatment fields in this photon-based rotational arc RT (**d**). Gross tumor volume: *red contour*; planning target volume: *green contour*; larynx: *blue contour*; spinal canal: *yellow contour*

Therefore, the actual dose delivered to the patient may differ from the planned dose owing to anatomical changes (Fig. 2). This phenomenon highly depends on treatment technique (e.g., field arrangement) and on radiation quality. Were the patient in Fig. 2 to be treated with protons (instead of photons), adaptation of the beam intensities would clearly be needed in order to ensure dose coverage and sparing of healthy tissues. Photon treatment plans are usually more robust, and thus adaption is often not necessary but still desirable so as to improve the sparing of normal tissue [12, 13].

For fast-changing intra-fraction motion, a different IGRT strategy is required than for slower and larger organ deformation. For breathing-induced tumor motion, which is to some extent predictable, gating [14] and tracking [15] techniques have been developed that often depend on predetermined motion models, where external motion of the patients’ surface correlates with internal tumor motion (with or without the use of fiducial markers). To prevent induction of systematic mismatches, the key point of these techniques is on-board image verification and updating of the motion models employed.

## Stereotactic RT rests on image guidance

Stereotactic radiotherapy (SRT) is a relatively recent development in RT although its origins date back to the 1950s. The basic principle of SRT is achieving extreme geometric accuracy of radiation dose delivery so that very small targets (tumors or intracranial arteriovenous malformations) can be treated with unprecedented high radiation doses causing ablation of the lesion—and severe damage if the target is missed. This principle thus highlights the particular importance of image guidance: In the early days of SRT, sufficient precision was only achievable by rigid fixation of the radiation device via a headframe fixed to the bony skull using screws. Obviously, before 3D and 4D imaging became available, SRT was mainly limited to the treatment of intracranial lesions.

With the introduction of CT and 3D and later 4D representation of the region to be irradiated, extending stereotactic RT to extracranial locations became possible. It has quickly become the guideline-recommended treatment of choice for patients with early stage non-small-cell lung cancer when the operative risk is deemed unacceptably high. In addition, stereotactic body RT (SBRT) is a very attractive option for patients with lung metastases, confined lesions in the adrenals, liver, or even elsewhere. Direct comparative studies of SBRT versus surgical removal of oligometastases are not available, but there is some evidence that SBRT may entail equal lesion control and survival for patients with lung oligometastases as can be obtained with surgery [16].

**Fig. 3:**
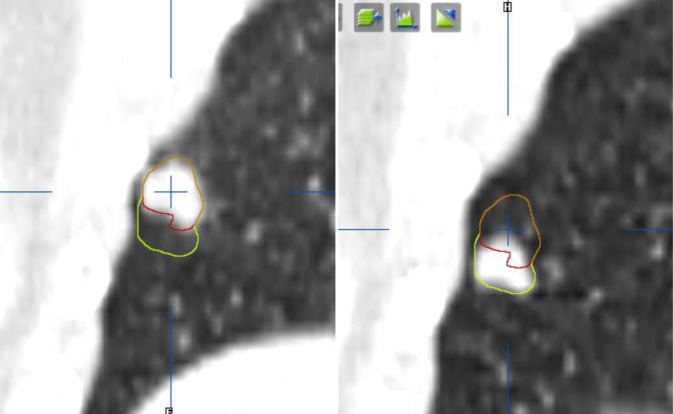
Moving lung tumor treated with stereotactic body radiotherapy. The *red contour* (tumor in expiration position) is extended to include the inspiration position, yielding an internal target volume (*yellow* plus *red contour*)

The essence of both intracranial and extracranial RT consists in administering very high doses per fraction to small target volumes, which in general only comprise gross tumor tissue with tight margins [17]. The treatment has a favorable toxicity profile and is very convenient for patients, as it is typically delivered in only one to five, sometimes eight to 12, fractions. Even for moving extracranial targets (lung, liver, adrenal), the safety (or uncertainty) margin around the lesion to be treated is in the millimeter range. This is made possible primarily owing to 4D-CT at treatment planning to tackle respiratory motion during treatment delivery [18], as it enables individualized definition of the volume to be irradiated (often called “internal target volume,” ITV; Fig. 3). Treatment itself is guided in turn by online imaging. On-board cone-beam CT either in 3D or even in 4D mode allows for adjustment of the treatment plan comprising multiple fields or arcs to the actual tumor position in the patient at every fraction of treatment delivery [19].

The second principle of SRT is to use steep dose gradients by prescribing the dose to the PTV encompassing the 50–80% isodose in order to therewith achieve a low-as-possible dose outside the target and accumulation of dose within the tumor [20, 21]. In contrast, conventional RT attempts to achieve dose profiles that are as homogeneous as possible within volumes irradiated, since typically, healthy but radiosensitive organs are situated within the clinical target volume that would otherwise be disproportionately harmed. In SRT, the target volume usually contains only tumor tissue, and therefore a higher dose within the target volume would only increase tumor control probability, which seems to be the case given the favorable clinical results of SRT. At first sight, it may sound paradoxical to decrease the dose outside the target by increasing the dose within the target [22]. This peculiarity was observed some time ago in rotational arc therapy [23], but has only recently been applied as a standard in clinical practice. Presently, treatment planning software as well as on-board CBCT during RT delivery render possible the individual optimization of dose delivery for every patient.

Classically, safe delivery of RT involves the definition of a safety margin to account for all uncertainties encountered between imaging for treatment planning and actual treatment delivery. This is typically achieved by adding some millimeters around the target volume, to result in a PTV. Improved on-board imaging reduces anatomical uncertainties and thus allows smaller PTV margins to be used. With more accurate information about, and registration of, tumor position at any time during treatment delivery, the dose could be (re)calculated real time during the respiratory cycle. That way, a high target coverage would be possible without simply adding a margin around the target to be treated. Evidently, this is completely dependent on the calculation speed of the treatment dose planning software and the accuracy of online imaging during treatment delivery (either CT or MRI based).

This principle, however, applies not only for SRT, but also for classic IMRT and will further enable us to escalate the dose within the target volume while at the same time reducing the dose in the OAR in the hope of therewith improving the probability to better control treated tumors.

## Adaptive RT: plan of the day concept

**Fig. 4:**
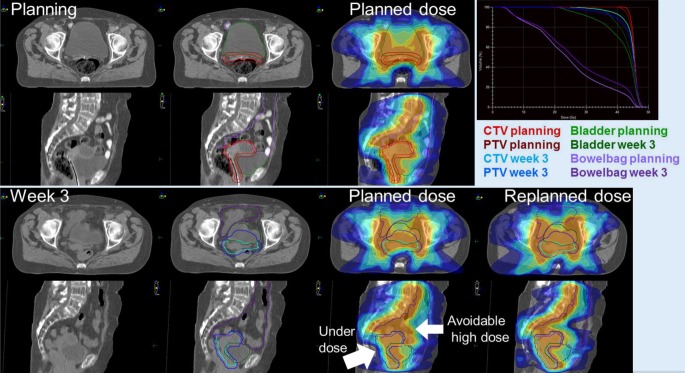
Example of how organ position can change as a function of different bladder and rectum filling. Planning computed tomography image and planned dose with filled bladder and rectum (*upper panel*); rescanning at week 3 (*lower panel*) with less filled bladder. Planned dose and recalculated dose distribution overlaid in color wash; *white arrows* depict underdose and avoidable high dose that were corrected by choosing a better suitable plan from the library (*lower panel*, re-planned dose). *CTV* clinical target volume, *PTV* planning target volume

For slower organ motion as, for example, in the pelvic area, dose distributions are ideally re-optimized just before each new treatment fraction because variable bladder or rectum filling can lead to substantial differences in dose distribution to the OAR and to the target. Since long calculation-optimization times, lack of automatic deformable image registration and delineation tools, and (dose)limits on high-frequency X‑ray-based imaging hamper full real-time IGRT, current IGRT methods try to overcome these limits by preparing pre-calculated libraries of plans [24]. These libraries are generated from pre-treatment planning CT scans with variable bladder filling (e.g., full bladder, empty bladder, and thereafter sequential CT scans after drinking 300 ml of water). On each day, the patient anatomy is determined on-board of the treatment machine with a cone-beam CT. Depending on the organ positions, the best fitting pre-designed dose distribution is then chosen from the library of plans. This way, conformity of the plan with the target volume is optimized. This process is shown in Fig. 4. Automatic planning approaches will reduce the clinical workload that is associated with the plan library design and re-planning, and will contribute to smaller volumes once it is possible to produce an even larger number of plans in a short time.

## MR-guided brachytherapy: accounting for anatomic changes during RT

The insertion of a brachytherapy-applicator causes non-rigid deformation of tissue. Additionally, during treatment the position of the applicator with respect to the target and OARs might change. For accurate and optimal dose distributions, MRI guidance increases target coverage and offers more control for OAR sparing, by imaging the full organ and applicator configurations, thus shaping the dose to the actual situation [25].

**Fig. 5:**
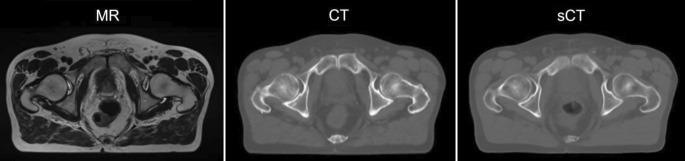
Synthetic computed tomography (*sCT*) calculated from magnetic resonance (*MR*) image only, using neural networks. This sCT can be used for radiation dose calculation just like an actual CT, which can in turn be skipped. (Courtesy of Lukas Fetty, Dept. of Radiotherapy, Medical University of Vienna)

In contrast to brachytherapy, where MR guidance can be considered as almost standard of care for treating cervical cancer [25], there is no consensus on how MRI is ideally integrated and adopted for external beam radiotherapy (EBRT). Since imaging in EBRT is used not only for defining targets and OAR but also for extracting attenuation information for dose calculation, some challenges need to be overcome when aiming at MRI-only-based EBRT, which would no longer need any planning CT scans (Fig. 5; [26–28]).

Geometric precision is of utmost importance in radiation oncology, and thus MR images need to be distortion corrected and geometric quality assurance needs to be established. The generation of CT-equivalent information from MRI for dose calculation and position verification in the treatment unit is prerequisite for using MRI-guided RT. The pixel intensity values in MRI are not associated with the attenuation properties of ionizing radiation with respect to the imaged material in the same way as CT data are. Where CT measures the attenuation of an X‑ray spectrum, the MR signal is related to the behavior of protons in a strong magnetic field. The pixel intensity value depends heavily on the sequence settings (e.g., T1, T2, usage of fluidic attenuation or fat suppression techniques) as well as on the micro-magnetic and chemical environment, which affects the relaxation time.

A rather challenging approach resulted in the construction of hybrid MRI plus LINAC treatment machines, where MRI and beam delivery share a common isocenter [29]. The three main technical hurdles—namely, magnetic interference, radiofrequency (RF) interference, and beam transmission through the magnet—could be overcome in the past few years and led to the design and construction of prototypes and clinically usable machines [30, 31].

## Image guidance and adaptation in particle RT

In today’s EBRT, ion and proton beams are considered the ultimate radiation type currently available. This can be explained by the inverted depth dose profiles of charged particles that are heavier than electrons, which enables the application of very conformal treatments with steep dose gradients around the tumor in all directions [32]. In addition, there may be biological advantages that depend on particle species. These physical and biological benefits will only translate into clinically relevant advantages if treatment planning and dose delivery are based on state-of-the art imaging technology including target definition and daily image guidance in- or outside the treatment room.

The traditional method of aligning patients at the light ion beam therapy facility was, until recently, based on orthogonal pairs of kV X‑ray images obtained every day in the treatment room. Only recently has CBCT technology become available for image guidance in particle therapy.

The use of scanned beam delivery, which superseded passive beam delivery for ion beam delivery, further motivates image guidance and treatment adaptations in particle therapy owing to the pronounced sensitivity of the particle range to anatomical changes (e.g., changes of structures filled with air or fluid, respectively) or setup errors. This also leads to the dilemma that treatment adaptations due to anatomical changes as mentioned earlier are much more frequently needed than in photon therapy. Roughly, about one third of all particle therapy patients, depending on treatment site, require at least one re-planning during their course of treatment (c.f., Fig. 2).

From a theoretical point of view, MR guidance is more important for particle than for photon RT, as particles are more sensitive to anatomical variations. Contrary to already commercially available solutions for MR-guided photon beam therapy, MR-guided proton beam therapy is still in a prenatal phase. There are basic questions that need to be addressed, such as the optimal field strength for MR-based image guidance, or obvious physical challenges that need to be overcome, as the charged proton beam will undergo deflections by the magnetic field.

## Functional imaging and dose painting

Anatomical imaging has significant limitations with regard to accurate delineation of the tumor. Tumor visualization (e.g., signal intensity) is based on differences in tissue density (CT) and magnetic properties (MRI), which are not specific to tumor tissue only. Prior surgery, RT or chemotherapy, or treatment-independent inflammatory processes may induce similar changes in density/signal intensity in normal tissues. Moreover, morphological imaging does not indicate whether the remaining tumor tissue is already sterilized or still viable and thus capable of forming recurrences after treatment. In recent years, new techniques for addressing these shortcomings, such as dynamic contrast-enhanced CT and MRI (DCE-CT/DCE-MRI) and diffusion-weighted MRI (DW-MRI), have been developed. Their potential role in RT treatment planning and treatment monitoring is the subject of intensive current research [33]. Positron emission tomography (PET) or single photon emission tomography (SPECT) in radiation oncology, in combination with CT or MRI, are rapidly expanding technologies [34]. Functional imaging visualizes biological processes and is supposed to have a higher sensitivity and specificity for tumor tissue, offering distinct and complementary advantages over purely anatomical imaging.

## Practical conclusion

- Imaging plays a decisive role in radiotherapy (RT) and together with improvements in computing power and algorithms is the major driver of progress in the field.
- The overarching aim is conforming the treatment dose to the tumor, in order to limit high radiation doses outside the volume requiring treatment as much as possible.
- While imaging for planning—pretreatment—has already reached a high level, the next step in improving RT will further address imaging during treatment delivery, resulting in an adaptation of the dose fluence as needed.
- Which imaging modalities will be optimal for which clinical situations will become evident in the years to come.
